# Incidence and Risk Factors of Serious Adverse Events during Antituberculous Treatment in Rwanda: A Prospective Cohort Study

**DOI:** 10.1371/journal.pone.0019566

**Published:** 2011-05-18

**Authors:** Natalie Lorent, Osee Sebatunzi, Gloria Mukeshimana, Jef Van den Ende, Joannes Clerinx

**Affiliations:** 1 Internal Medicine Department, Centre Hospitalier Universitaire de Kigali, Kigali, Rwanda; 2 Clinical Sciences Department, Institute of Tropical Medicine, Antwerp, Belgium; McGill University, Canada

## Abstract

**Background:**

Tuberculosis (TB) and TB-human immunodeficiency virus infection (HIV) coinfection is a major public health concern in resource-limited settings. Although TB treatment is challenging in HIV-infected patients because of treatment interactions, immunopathological reactions, and concurrent infections, few prospective studies have addressed this in sub-Saharan Africa.

In this study we aimed to determine incidence, causes of, and risk factors for serious adverse events among patients on first-line antituberculous treatment, as well as its impact on antituberculous treatment outcome.

**Methods and findings:**

Prospective observational cohort study of adults treated for TB at the Internal Medicine department of the Kigali University Hospital from May 2008 through August 2009.

Of 263 patients enrolled, 253 were retained for analysis: median age 35 (Interquartile range, IQR 28–40), 55% male, 66% HIV-positive with a median CD4 count 104 cells/mm^3^ (IQR 44–248 cells/mm^3^). Forty percent had pulmonary TB, 43% extrapulmonary TB and 17% a mixed form. Sixty-four (26%) developed a serious adverse event; 58/167 (35%) HIV-infected vs. 6/86 (7%) HIV-uninfected individuals. Commonest events were concurrent infection (n = 32), drug-induced hepatitis (n = 24) and paradoxical reactions/TB-IRIS (n = 23).

HIV-infection (adjusted Hazard Ratio, aHR 3.4, 95% Confidence Interval, CI 1.4–8.7) and extrapulmonary TB (aHR 2, 95%CI 1.1–3.7) were associated with an increased risk of serious adverse events. For TB/HIV co-infected patients, extrapulmonary TB (aHR 2.0, 95%CI 1.1–3.9) and CD4 count <100 cells/mm3 at TB diagnosis (aHR 1.7, 95%CI 1.0–2.9) were independent predictors. Adverse events were associated with an almost two-fold higher risk of unsuccessful treatment outcome at 6 months (HR 1.89, 95%CI 1.3–3.0).

**Conclusion:**

Adverse events frequently complicate the course of antituberculous treatment and worsen treatment outcome, particularly in patients with extrapulmonary TB and advanced immunodeficiency. Concurrent infection accounts for most events. Our data suggest that deterioration in a patient already receiving antituberculous treatment should prompt an aggressive search for additional infections.

## Introduction

Tuberculosis remains one of the deadliest infectious diseases in the developing world and the human immunodeficiency virus (HIV) is its single most important risk factor [1]. Tuberculosis drug related adverse reactions such as hepatitis, peripheral neuropathy, gastro-intestinal intolerance and skin rashes commonly occur [2]. They can cause significant morbidity and therefore compromise adherence, eventually contributing to treatment failure, relapse or emergence of resistant strains [3]. Likewise, tuberculosis treatment interruptions, which are required if standard tuberculosis treatment is not tolerated, may result in suboptimal treatment response. Adverse drug reactions have been associated with older age, female sex, and HIV [2], [4], [5]. In addition, malnutrition, pre-existent liver disease, genetic factors, use of concomitant drugs and alcohol are risk factors more specifically related to liver toxicity [6]–[9]. Besides drug-related events, immunological reactions and concurrent infections can play a role in the clinical deterioration of patients on antituberculous treatment, particularly in the HIV-infected [10]. In sub-Saharan Africa few prospective studies have dealt with clinical AEs during antituberculous treatment and their impact on treatment outcome [11], [12].

In this study we aimed to determine incidence, causes of and risk factors for serious AEs among patients on first-line antituberculous treatment. We also examined its impact on antituberculous treatment outcome. Preliminary results have been previously presented at the Union's World Conference [13].

## Methods

### Ethical review

This was an observational cohort study. Patient care followed standard diagnostic and treatment procedures of the department of Internal Medicine of the CHUK. Written informed consent was obtained from all participants involved in the study.The study was reviewed and approved by both institutional review boards of the Centre Hospitalier Universitaire de Kigali and the Institute of Tropical Medicine in Antwerp, as well as by the Rwanda National Ethics Committee.

### Study site and setting

We conducted a prospective cohort study at the Centre Hospitalier Universitaire de Kigali (CHUK) from May 2008 through January 2010. The CHUK with its 500-bed capacity is the largest of the four public teaching hospitals in Rwanda.

### Patient inclusion and follow-up

We enrolled all adult patients (aged ≥21 years) - both inpatient and outpatient – who had started first-line TB treatment for newly diagnosed TB at the Internal Medicine Department of the CHUK. We excluded prisoners, patients residing outside greater Kigali, and patients who were unable or unwilling to provide written informed consent.

Patients were seen at regularly scheduled visits at 2, 4, 8, 12 and 24 weeks of TB treatment, and were encouraged to return at any time if new symptoms arose during therapy. Patients who missed a scheduled visit were contacted by phone or, if this was unsuccessful, through a TB clinic visit by the study nurse.

Data recorded at intake included demographic information (sex, age, civil status, educational level, use of tobacco, alcohol, and traditional medicine) and clinical data (TB presentation, data on previous TB treatment, HIV serostatus, CD4 within 3 months before or at TB diagnosis, antiretroviral and cotrimoxazole use, temperature and body mass index). Full blood count, liver enzymes, and serum creatinin were determined at baseline, and follow-up visits. New onset symptoms or signs were documented, as well as the interval from antituberculous treatment initiation.

When patients' condition deteriorated, the cause of it was investigated through additional diagnostic tests such as urine, stool, cerebrospinal fluid, pleural fluid and ascites analysis, blood culture, chest radiography, abdominal ultrasound when deemed necessary by the treating physician. Diagnosis at clinical deterioration and outcome at 24 weeks were determined for all patients. Since the study site is a tertiary referral hospital, all patients are referred to their nearest health centre for directly observed therapy. The study nurse ascertained the final outcome through contacting the referring health centres and through consultation of the national TB register. Outcome of patients that could not be traced was labelled as unknown.

### TB/HIV diagnosis and treatment

Patients were defined as having microbiologically-confirmed TB if at least one biological specimen was positive for acid-fast bacilli. Culture for *Mycobacterium tuberculosis* was done on request when drug resistance was suspected. Smear-negative or extra-pulmonary TB was based on standard WHO case definitions [14]. Initiation of TB treatment in those cases required assessment by at least two senior physicians from the department, experienced in TB care.

According to the national protocol, patients with a new diagnosis of TB receive 6 months of antituberculous treatment consisting of 2 months of rifampin (R), isoniazid (H), ethambutol (E) and pyrainamide (Z) followed by 4 months of R and H (2RHEZ/4RH). In the retreatment regimen streptomycin (S) is added to the intensive phase: 2SRHEZ/1RHEZ/5RHE.

First-line ART consists of two nucleoside reverse transcriptase inhibitors (stavudine or zidovudine or tenofovir plus lamivudine) and a non-nucleoside reverse transcriptase inhibitor (nevirapine or efavirenz). National guidelines recommend initiation of ART for all extrapulmonary TB regardless of CD4 count, and pulmonary TB with CD4 count <200 cells/mm3 within two to eight weeks after starting of antituberculous treatment. In case of pulmonary TB with CD4 counts between 200 and 350 cells/mm^3^ initiation of ART is recommended after the intensive phase of antituberculous treatment. Preferably, all TB patients are either switched to or started on an efavirenz-based regimen because of potential drug interactions of rifampin with nevirapine.

### Definitions

A serious AE was defined as an acute clinical deterioration occurring after the onset of antituberculous treatment, requiring any change in treatment and/or hospitalisation. We subdivided the causes of AEs into: 1) toxic drug reactions, 2) concurrent infection (or neoplasm), 3) antituberculous treatment failure, 4) paradoxical reactions or paradoxical TB-associated immune reconstitution inflammatory syndrome (TB-IRIS).

Data from a preliminary study revealed drug-induced hepatitis was common while the prevalence of viral hepatitis was very low (N. Lorent, personal communication). Drug induced liver toxicity was defined as symptomatic elevation of serum transaminases (more than three times the upper limit of normal) and/or jaundice during antituberculous therapy, after exclusion of other apparent causes. Concurrent infection (proven or suspected) was defined as an acute febrile illness that occurred after TB diagnosis and that presented with a sepsis-like syndrome manifested by two or more of the following conditions: heart rate >90/min, respiratory rate >20 breaths/min, temperature >38°C or <36°C, white cell count >12 000 cells/mm^3^ or <4 000 cells/mm^3^ [15]. We defined paradoxical TB-IRIS according to consensus clinical case definition for resource-limited settings [16], adapted to the diagnostic capacities of our setting. A paradoxical reaction was classified as probable when 1) an initial favorable response to antituberculous treatment was followed by 2) new onset or recrudescence of TB manifestations and 3) at least a minimum diagnostic work-up had been done to exclude potential alternative diagnosis such as poor adherence, drug reactions, and concurrent infection. A paradoxical reaction was classified as possible if the abovementioned third condition had not been fulfilled. Causes of severe AEs could be multiple. They were determined by consensus of the first and second author based on the available clinical information. Only the first clinical event was taken into account.

Unsuccessful treatment outcome includes treatment failure (excluding three patients with confirmed alternative diagnosis), default, death, or lost to follow-up at final evaluation.

### Statistical analysis

Patients' demographic and clinical characteristics were described in terms of percents, medians and interquartile ranges (IQR). We calculated frequencies and proportions and used Chi2-test (or Fisher's exact) and Student's t-test (parametrical) or Wilcoxon rank-sum (non-parametrical data) to compare proportions and medians, respectively. Cut-off values for dichotomous variables were based on their medians.

We used Kaplan-Meier estimates to evaluate the cumulative probability of acute clinical deterioration over 24 weeks on TB treatment. Accounting for the variable time and for multiple factors potentially affecting their occurrence, we used Cox proportional hazards multivariate regression to estimate adjusted hazard ratios (aHR), and their 95% confidence intervals (CI) for risk factors for serious AEs. The proportional hazard assumption was tested graphically - by plotting the log (cumulative hazard) *vs* follow-up time - and formally using Schoenfeld residuals. Well-known confounding factors, and factors found to be associated with the occurrence of an AE with a p-value less than 0.05 in the univariate model were considered as potential covariates in the multivariate models. Hepatis B surface antigen was not included in the modeling given the high number of missing results (118/253). Patients were censored at the occurrence of death, loss to follow-up or clinical evaluation on week 24, whichever occurred first.

To investigate the association between adverse events and death we used Cox regression with split follow-up time at the occurrence of a serious adverse event.

A two-sided p-value<0.05 was considered significant. All analyses were performed using STATA® software version 10.0 (College Station, Texas, USA).

## Results

**Figure 1:**
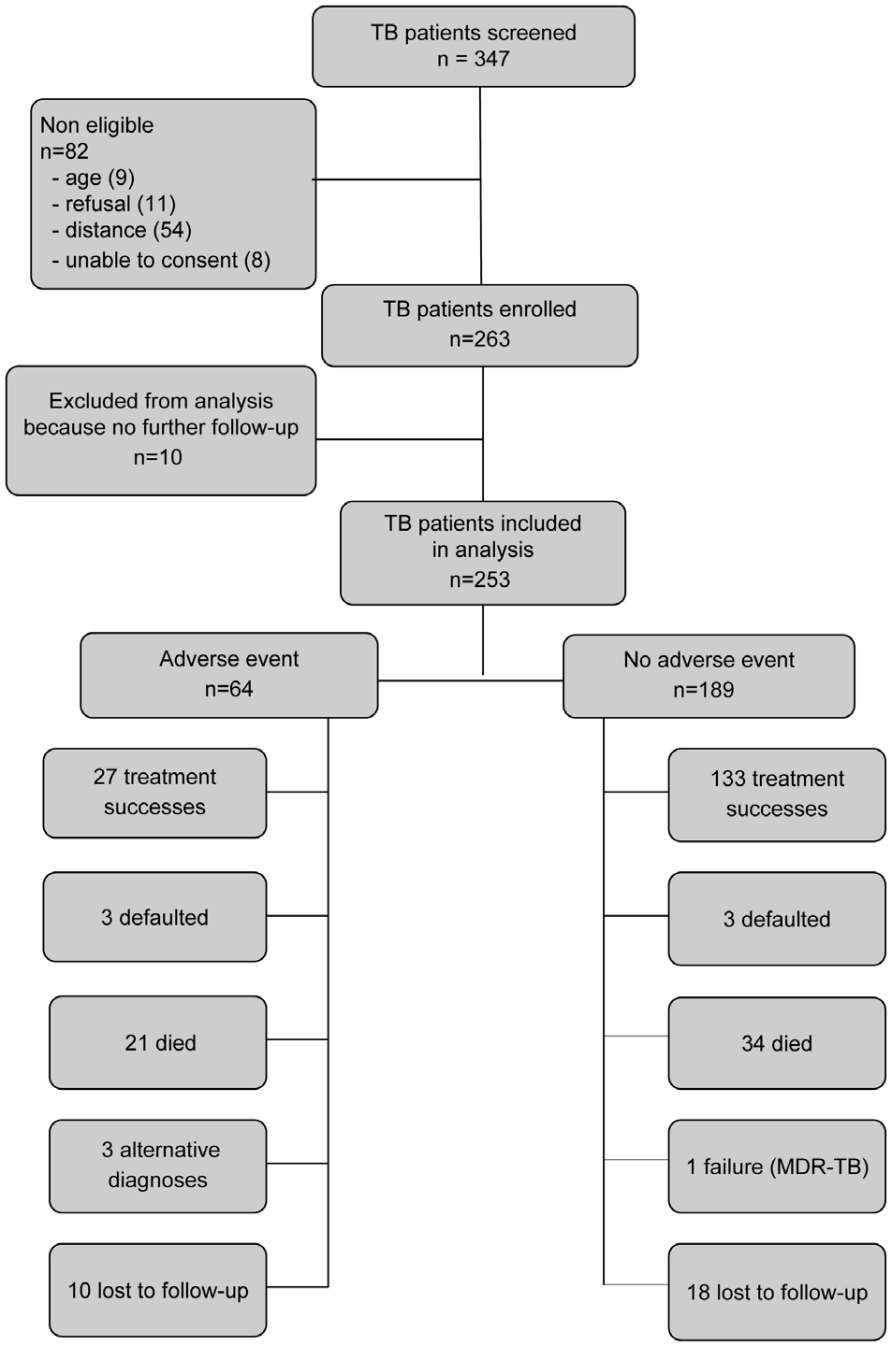
Flow diagram of all adult patients who started antituberculous treatment from 1 May 2007–31 July 2009.

Between May 2008 and August 2009, 347 patients started first-line antituberculous treatment for presumptive or confirmed TB. Acid-fast bacilli smears were positive in 78/145 (54%) patients. Figure 1 is showing a flow diagram of all assessed TB patients. Eighty-two patients were not eligible: 54 due to residence outside the 20 km radius from the hospital, 11 refused to participate, and 9 were less than 21 years of age. Of the 263 enrolled, we excluded from analysis ten patients who did not return for any follow-up visit and who could not be traced for the whole study duration.

Baseline characteristics of the remaining 253 patients are shown in Table 1. TB/HIV co-infected patients were of slightly older age, were more likely to have smear-negative and/or extrapulmonary TB, low baseline hemoglobin levels, to have had prior TB, and to require hospital admission.

**Table 1:** Baseline characteristics of 253 tuberculosis patients by HIV status in a tertiary care hospital in Rwanda.

	HIV positive	HIV negative	p-value
	n = 167	n = 86	
	n (%)	n (%)	
**Demographics**			
Age in years, median (IQR)	36 (30–42)	32 (24–36)	0.0004
Female sex	76 (45.5)	39 (45)	ns
Married	77 (46)	32 (37)	ns
No formal education	17 (10)	9 (10)	ns
Unemployed or student	44 (26)	44 (51)	<0.0001
Alcohol abuse	124 (73)	48 (56)	0.003
Smoking, active	63 (38)	21 (24)	0.033
Hospital admission	130 (78)	23 (27)	<0.0001
**Clinical**			
Body mass index^a^, median (IQR)	17,9 (16,0–20,2)	18,9 (16,8–20,4)	ns
Hemoglobin (g/dL), median (IQR)	9,9 (8,4–12)	13,1 (11,6–14,5)	<0.0001
Site of TB^b^			
Pulmonary	54 (32)	48 (55)	0.001
Extrapulmonary	77 (46)	31 (36)	
Pulmonary and extrapulmonary	36 (22)	7 (8)	
Smear positivity (n = 144)^c^	39 (44)	39 (71)	0.002
Positive hepatitis B surface antigen (n = 135)^d^	6 (7)	1 (2)	ns
**Previous TB treatment**	25 (15)	4 (5)	0.015
**Fluconazole treatment** e	32 (19)	0 (0)	<0.0001
**Traditional drugs** e	12 (7)	7 (8)	ns
**Severe adverse event**	58 (35)	6 (7)	<0.0001

a Body mass index is defined as the weight in kilograms divided by the square of the height in meters.

b TB can affect multiples sites in one patient.

c Smear-positivity from any biological specimen, including sputum, lymph node aspirate, etc.

d Hepatitis B surface antigen positive in 6/87 hiv-infected and 1/48 hiv-uninfected persons.

e within one week prior to admission.

p-value<0.05; IQR = interquartile range.

One hundred sixty-seven (66%) persons were HIV-seropositive. Median CD4 count was 104 cells/mm^3^ (IQR 44–248 cells/mm^3^). Sixty five (39%) patients were receiving ART at initial TB diagnosis. Of the 97 patients who qualified for ART at study entry, 55 (33%) were started on ART within a median of 31 (IQR 14–50) days; of whom 43 (78%) during the intensive phase of antituberculous treatment. Cotrimoxazole preventive therapy or dapsone in case of sulfonamide allergy was given to 95% of the patients.

Sixty-four patients (26%) developed a serious AE, 35% (58/167) in HIV-infected versus 7% (6/86) in HIV-uninfected individuals. The commonest were concurrent infection (n = 33), drug-induced hepatitis (n = 24) and paradoxical reactions/paradoxical TB-IRIS (n = 20) as shown in Table 2.

**Table 2:** Causes of serious clinical events during 6 months follow-up on antituberculous treatment in 167 HIV-infected and 86 HIV-uninfected individuals.

	HIV-infected (n = 167)	HIV-uninfected (n = 86)
**Total number of serious adverse events**		
**in number of patients (pat)**	**77 events in 58 pat**	**6 events in 6 pat**
**Concomitant infection**	**31 (19%)**	**2 (2%)**
Confirmed infection^a^	9	-
Suspected infection^b^	22	2
**Drug-induced liver toxicity**	**22 (13%)**	**2 (2%)**
**Paradoxical reactions/TB IRIS** c	**21 (13%)**	**1 (1%)**
probable	12	1
possible	9	-
**Treatment failure (multidrug-resistant TB)**	**-**	**1 (1%)**
**Miscellanous**	**5 (3%)**	**-**
Suspected lymphoma	2	-
Suspected Kaposi's sarcoma	1	-

a illnesses with identified etiologic agent were: *Klebsiella pneumoniae* bacteremia (2), *Staphylococcus aureus* bacteremia (1), *Klebsiella pneumoniae* urosepsis (2), cryptococcal meningitis (1), zona ophtalmica (1), Plasmodium falciparum malaria (1), *Escherichia coli* dysentery (1).

b in most cases no etiologic agent was identified including ilnesses such as hepatic abscess, enteritis, cholangitis, bronchopneumonia, urosepsis.

c immune reconstitution inflammatory syndrome (IRIS) is used according to consensus case definitions for resource-limited countries [16]; paradoxical reaction is the term used for HIV-uninfected persons.

In only 9/32 cases a causative organism of concurrent infection was isolated (Table 2). Drug-induced liver toxicity developed in 22 HIV-infected and two HIV-uninfected individuals, all of whom were symptomatic (nausea, vomiting, anorexia, abdominal pain and/or jaundice). Only one case of liver toxicity occurred in an individual with chronic hepatitis B co-infection. Paradoxical reactions occurred in 21 HIV-infected individuals (12 probable and nine possible) and in only one HIV-uninfected person. Primary multi-drug resistant TB was confirmed in one HIV-uninfected individual. Alternate diagnoses including lymphoma and Kaposi's sarcoma were suspected in three patients.

**Figure 2:**
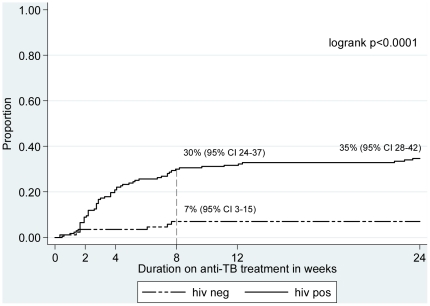
Kaplan-Meier estimates of serious adverse events by HIV-status for 253 tuberculosis patients. The estimated cumulative probability to develop an adverse event was significantly higher in HIV/TB co-infected patients: 20.9% (95% CI 15.5–27.9) within the first month of antituberculous treatment (vs. 3.0% (95% CI 1–10) in HIV-uninfected) and up to 29.9% (95%CI 23.6–37.5) at two months of treatment (vs. 6.9% (95% CI 3.2–14.9) in HIV-uninfected).

The median time to develop an AE was 23 days (IQR 14–51). As shown in Figure 2, HIV/TB co-infected patients are at a substantially higher risk to develop an AE within the first two months of antituberculous treatment.

TB treatment was interrupted in 22/64 (34%) patients who developed an event, 9% of the total cohort. In 17/22 individuals this was temporarily and all drugs were successfully reintroduced within 4 weeks.

As shown in Table 3, HIV-infection (aHR 3.4, 95% CI 1.4–8.7) and extrapulmonary TB (aHR 2, 95%CI1.1–3.7) were the only two independent risk factors that remained associated with AEs after multivariate analysis.

**Table 3:** Predictors of serious adverse events for all 253 tuberculosis patients in univariate and multivariate analysis.

Variable	unadjusted HR	95% CI	p-value	adjusted HR	95% CI	p-value
Age <35 years	0.97	0.59–1.58	ns	-		
Female sex	1.25	0.77–2.04	ns	-		
Hospital admission	3.70	1.93–7.08	<0.0001	1.76	0.85–3.67	ns
Extrapulmonary tuberculosis	2.74	1.52–4.97	0.001	**2.04**	1.12–3.72	0.020
Prior tuberculosis history	1.31	0.65–2.64	ns	-		
Body mass index <18.5 kg/mm^2^	2.01	1.18–3.41	0.010	1.35	0.78–2.34	ns
Hemoglobin <11 g/dL	2.34	1.40–3.90	0.001	1.17	0.70–1.97	ns
HIV co-infection	5.81	2.51–13.47	<0.0001	**3.42**	1.35–8.67	0.009
Smoking	1.06	0.63–1.77	ns	**-**		
Alcohol intake	1.02	0.60–1.73	ns	**-**		

HR: hazard ratio, Cox proportional hazard regression analysis; 95% CI: 95% confidence interval; p-value<0.05.

In the 167 TB/HIV co-infected patients, extrapulmonary TB (aHR 2, 95%CI 1.1–3.9) was the only factor independently associated with an increased risk of developing a serious AE (Table 4). No significant associations were found between developing a serious AE and sex, age, previous TB treatment, and ART during TB treatment.

**Table 4:** Predictors of serious adverse events for 167 HIV-infected tuberculosis patients in univariate and multivariate analysis.

Variable	unadjusted HR	95% CI	p-value	adjusted HR	95% CI	p-value
Age <35 years	1.62	0.97–2.71	ns	1.41	0.83–2.40	ns
Female sex	1.30	0.78–2.17	ns	-		
Smoking	0.91	0.53–1.55	ns	-		
Alcohol intake	0.78	0.44–1.37	ns	-		
Hospital admission	2.04	0.97–4.30	ns	1.44	0.65–3.19	ns
Extrapulmonary tuberculosis	2.12	1.12–4.00	0.020	**1.99**	1.05–3.76	0.036
Prior tuberculosis history	1.05	0.51–2.13	ns	-		
Body mass index <18.5 kg/mm2	1.50	0.86–2.61	ns	-		
Hemoglobin <11 g/dL	1.40	0.80–2.45	ns	-		
CD4 count <100 cells/mm3	1.71	1.01–2.90	0.044	1.55	0.90–2.70	ns
Use of antiretroviral treatment	1.36	0.73–2.52	ns	-		

HR: hazard ratio, Cox proportional hazard regression analysis; 95% CI: 95% confidence interval; p-value<0.05.

Median follow-up time among patients who survived was 175 days (IQR 161–180). Treatment outcomes were ascertained for 225 persons. Of 28 (11.1%) patients who had been transferred out no information could be found in the hospital's TB register. Hundred and sixty (63.2%) patients were successfully treated. Six (2.4%) defaulted, of whom 4 refused to continue antituberculous treatment following an event. One confirmed multidrug-resistant TB patient was classified as treatment failure. Treatment interruption was decided upon by the medical staff after histological confirmation of an alternative diagnosis in 3 patients (1 non Hodgkin lymphoma, 1 Kaposi's sarcoma, 1 lung cancer).

AEs were associated with an almost two-fold increased risk of unsuccessful treatment outcome after 6 months of antituberculous treatment (HR 1.9, 95%CI 1.3–3.0, p = 0.002), as well as death (HR 9.0, 95% CI 4.7–17.3, p<0.0001). Exploring the impact of the three commonest causes of serious AEs in this, only (proven or confirmed) concurrent infection increases more than two-fold the risk of an unsuccessful outcome (aHR 2.2, 95%CI 1.3–3.7, p = 0.005).

Fifty-five (21.7%) patients died, all but 2/55 were (re)hospitalized and 48/55 were HIV infected. In thirty-four patients who did not experience a serious AE, death occurred at a median of 11 days (IQR 8–18) after a gradual deterioration of the general condition without development of new clinical signs or symptoms. Seventeen deaths (6.7%) were attributed to a serious AE and 12/17 patients presented a sepsis-like syndrome at the time of death.

## Discussion

In the present study, serious adverse events frequently complicated the course of antituberculous treatment, particularly in HIV-infected individuals. These events occurred despite successful integration of TB and HIV services, as reflected by HIV testing and counselling for all TB patients, 95% coverage with cotrimoxazole preventive therapy, and attempts to initiate ART in concordance with national guidelines.

Overall, more than a quarter (26%) of the patients developed a serious clinical event. In a study by Pepper, et al., 40% of the TB patients experienced clinical deterioration during 24 weeks of follow-up [12]. However, they used a broader definition of clinical deterioration. They included both all symptomatic worsening, no matter which grade of severity, and failure to stabilise within 24 weeks of antituberculous treatment; whereas we mainly focused on predefined conditions such as concurrent infections, liver toxicity, paradoxical reactions, and treatment failure, based on the results of a preliminary study held in our department [17].

HIV-infection was the most important risk factor for developing a clinical event on antituberculous therapy, as has been extensively reported by others [12], [18]–[20]. This correlation is due to the increased risk of drug interactions/toxicity, infections, and TB-IRIS in HIV-infected individuals.

A substantial part of these AEs resulted from - presumed or confirmed - concurrent infection, reflecting the profound immune suppression by HIV (and TB) [12], [19],[21]

Often fever in the course of antituberculous treatment is not thoroughly investigated as it is attributed to the active TB disease process rather than to a concurrent infection. In low-resource countries such as Rwanda, diagnosing a concurrent infection in a TB patient is challenging due to indiscriminate use of antibiotics and limited diagnostic tools. Furthermore, if no causative organism is found, distinguishing (presumed) concurrent infection from drug hypersensitivity or paradoxical reaction/immune reconstitution syndrome may be problematic [16].

Diagnosis of IRIS remains difficult in sub-Saharan Africa, even with the prospective use of TB-IRIS case definitions for resource-limited settings [16]. In previous reports from low-income settings the proportion of patients developing TB-associated IRIS ranged from 8–16% [11], [22]–[25]. In the present study, TB-IRIS occurred in 9% of the patients, despite the rather short time interval between the initiation of antituberculous drugs and ART and the low baseline CD4 count, two recognised risk factors [23], [26]–[28].

Consistent with findings from Botswana, drug-induced liver toxicity occurred in 9% of our patients [29]. However, studies on hepatotoxicity in TB and HIV co-infected patient populations from Tanzania, South-Africa and Malawi report much lower incidence rates ranging from 0.9% to 2% [5], [30], [31]. We did active laboratory surveillance for liver disease, potentially overestimating its occurrence. This is nevertheless unlikely since all hepatic events were symptomatic. TB-IRIS with hepatic involvement may be mistaken for hepatotoxicity [32]. Besides HIV infection, we were unable to identify other potential risk factors of hepatotoxicity such as traditional medicines, fluconazole [33], ART, and hepatitis B surface antigen carrier state given the limited number of events.

Drug-induced liver toxicity was reversible when antituberculous treatment was temporarily withdrawn, and did not result in an overtly adverse outcome.

Although well below international targets [34], a treatment success rate of 63% is not surprising for our setting. We attended a selected patient population of predominantly in-patients, with a high rate of TB and HIV co-infection and advanced disease which may be due to late presentation to and late referral from a health centre, factors well known to worsen TB treatment outcome [35]–[38]. The high early mortality rate is consistent with data from Malawi [36].

Concurrent infection in the course of antituberculous treatment was associated with a more than two-fold increased risk of unsuccessful treatment outcome, including death [38]. Delay in diagnosis and appropriate treatment of sepsis [39], as well as lack of screening for opportunistic infections in HIV-infected individuals starting ART contribute to poor treatment outcome [40]. Therefore, deterioration in patients receiving antituberculous treatment should prompt an aggressive search for additional infections. In low-resource settings with limited diagnostic capacity this is far from evident.

Although we identified only four patients with bacteraemia, this may well be underestimated. Sepsis with bacteraemia is common in Africa and is fuelled by the HIV-pandemic [41]. We were unable to investigate to which extent disseminated mycobacterial disease has been the cause of clinical worsening, but in a recent systematic review on community-acquired bloodstream infections in Africa *Mycobacterium tuberculosis complex* accounted for 30% [41].

Limitations of our study basically relate to its design. Studies within the routine care of a busy single centre tertiary care centre suffer referral bias. Consequently the extent of the problem of AEs on antituberculous treatment cannot be precisely determined and our findings may not be transposed to other settings where HIV-1 associated TB is less common. By including outpatients we attempted to limit referral bias. Future studies of multicentric design may better address this issue.

Diagnostic work-up of an AE may have been incomplete because this was an observational study where the decision to further investigate was left at the physicians' discretion, and because diagnostic tools are lacking. However, these conditions very well reflect real life situation, even at tertiary care level in many sub-Saharan African countries.

In summary, AEs frequently complicate the course of antituberculous treatment and worsen treatment outcome. Concurrent infection accounts for most events. HIV infected patients with advanced immunodeficiency and extrapulmonary TB are more likely to develop clinical deterioration. Our data suggest that deterioration in a patient already receiving antituberculous treatment should prompt an aggressive search for additional infections.
